# Protective effect of caffeine against high sugar-induced transcription of microRNAs and consequent gene silencing: A study using lenses of galactosemic mice

**Published:** 2013-02-25

**Authors:** Shambhu D. Varma, Svitlana Kovtun

**Affiliations:** 1 Department of Ophthalmology and Visual Sciences, University of Maryland School of Medicine, Baltimore, MD; 2 Department of Biochemistry and Molecular Biology, University of Maryland School of Medicine, Baltimore, MD

## Abstract

**Purpose:**

Previous studies have shown that caffeine prevents the formation of cataracts induced by a high-galactose diet and consequent oxidative stress. The objective of this study was to investigate if this protective effect is reflected in the attenuation of the transcription of microRNAs (miRNAs) known to induce apoptosis and cell death by gene silencing.

**Methods:**

Young CD-1 mice were fed either a normal laboratory diet or a diet containing 25% galactose with or without 1% caffeine. One week later, the animals were euthanized, and the lenses isolated and promptly processed for RNA isolation and subsequent preparation of cDNAs by reverse transcriptase reaction. Mature miRNA (miR)-specific cDNAs were then quantified with PCR in a 96-well microRNA-specific cassette using an ABI7900HT PCR machine.

**Results:**

As expected from previous studies, the lenses were positive for all 84 miRs corresponding to the miRNA probes present in the cassette wells. However, the levels of at least 19 miRs were significantly elevated in galactosemic lenses compared to those in the normal lenses. The majority are proapoptotic. Such elevation was inhibited by caffeine. This has been demonstrated for the first time.

**Conclusions:**

Since aberrant elevation of miRNAs silences various genes and consequently deactivates protein translation, and since caffeine downregulates such aberration, the beneficial effect of caffeine could be attributed to its ability to suppress elevation of toxic miRs and consequent gene silencing.

## Introduction

MicroRNAs (miRNAs), noncoding and approximately 22 nt long ribonucleic acid molecules [1], control the functions of several genes under normal and diseased states [2]. MicroRNAs do so primarily by hybridization with nearly complementary base sequences in the 3′ untranslated regions of messenger RNAs (mRNAs) and consequently disabling them from performing their protein translational function [3]. Thus, mature microRNAs (miRs) function as important agents of gene silencing [4]. Since complete Watson and Crick complementarity is not required for hybridization of small nucleic acid molecules, the effect of miRs on gene silencing and subsequent physiologic effects could be more diverse than ordinarily expected [2]. In addition to the direct inhibitory effect on protein translation, such hybridization can also lead to destabilization of the mRNA itself and its eventual degradation. Physiologically, the resulting inhibition of protein translation is expected to be partial and total, depending upon the number of available miR molecules, as well as the number of available mRNA targets. In addition to targeting mRNAs, miRs have also been reported to hybridize with complementary sequences in the chromatin structure. This can also cause gene silencing at the transcriptional level [5-7], limiting the transcription of mRNAs. Hybridization with mRNAs involved in the biogenesis of enzymes and cofactors involved in reactions causing methylation and acetylation [8-11] can also lead to generational and intergenerational epigenetic effects. Overexpression of miRs is therefore generally a toxic event, except in situations where the overexpression can be used to suppress the translational activity of mRNAs producing toxic proteins or enzymes, as is the case in certain cancers [12-14].

In addition to cancer, miR expression becomes deregulated in many other pathophysiological conditions, such as with the development of certain cardiac myopathies including cardiac hypertrophy and abnormalities in rhythmic amplitude and periodicity [15-17], neural malfunctions such as with Alzheimer disease and senile dementia [18,19], and diabetes [20,21]. Redox deregulation and consequent oxidative stress are cardinal features of most of these conditions, including diseases related to the loss of vision with aging and diabetes [22-27]. The high prevalence and severity of visual impairment in the diabetic population are strongly correlated with the degree of hyperglycemia [23]. This is evident form the delay in the development of vision loss achieved by controlling blood glucose levels toward normal levels [28]. That the high sugar level may play a significant role in visual complications is also apparent from the development of cataracts in animals maintained on diets containing high amounts of other sugars such as galactose [29], arabinose [30], and xylose [31,32], in addition to diabetes [33-36]. Galactose has also been reported to cause abnormalities in retinal vasculature similar to that in diabetes [37,38]. However, information on the possible implications of aberrations in miR transcription and gene silencing induced by sugar-induced oxidative stress by high sugars remains limited. Such a possibility is strongly indicated by a recent report showing elevation in the transcription of several apoptotic miRs in the lenses of mice fed a high-galactose diet. That this elevation could be caused by oxidative stress was strongly indicated by the corrective effect of pyruvate, known to inhibit oxidative stress and cataract formation [39-42]. The increase in apoptotic miRs in galactosemic lenses was substantially attenuated in animals fed a diet mixed with pyruvate. Pyruvate also reverses oxidative stress–induced inhibition of glycolysis in the retina [43]. These studies on the possible enhancement of the transcription of toxic miRs caused by sugar-induced oxidative stress and its possible prevention by antioxidants have now been further examined using caffeine, a nutraceutical-derived compound known to act as a potent antioxidant [44,45] as well as inhibit apoptosis and cataract formation induced by a high-galactose diet [46,47]. We have now observed that the overexpression of most miRs noted in the galactosemic lens is significantly inhibited by adding caffeine to the diet. These findings could also be useful in explaining the lower incidence of cataracts in groups of persons consuming relatively higher levels of caffeine through coffee drinking (FAO drinks) [48,49].

## Methods

### Materials

CD-1 mice were obtained from Harlan Lab Inc. (Indianapolis, IN). Reagents for RNA isolation, cDNA preparation, PCR amplification of the cDNAs, and the miRNA-finder array were obtained from SA Biosciences Corporation (Frederick, MD). Routine chemicals were obtained from Sigma-Aldrich (St. Louis, MO).

### Methods

Mice weighing about 20 g were fed owdered rodent chow (controls- Harlan Farm diet # 2018SX; Indianapolis, IN) or chow mixed with galactose to the 25% level or mixed with 25% galactose plus 1% caffeine. The dietary regimen was maintained for 7 days. The animals were labeled as A, B and C and fed concurrently with the respective diet. They were also scarified, and lens isolated at the same time to avoid any technical and diurnal variation. The numbers of animals in the individual groups were 2, 3 and 2 respectively, giving at least 4, 6 and 4 lenses in each group for analysis.The blood galactose level was 4±0.5 mm in both groups of galactosemic animals. The dietary regimens were maintained for only 7 days to minimize the extent of cataractous changes in the lens and consequent leakage of material. Subsequently, the animals were anesthetized with a ketamine/xylazine mixture (6 mg/100 g ketamine and 0.75 mg/100 g xylazine) and quickly euthanized by CO_2_ inhalation. The eyes were then promptly enucleated and dissected to isolate intact lenses atraumatically. The isolated lenses were immediately frozen by immersion in liquid nitrogen and then immediately processed for miRNA-enriched RNA isolation using a Qiagen RNeasy reagent kit (cat no. 217,004; Valencia, CA), following the manufacturer’s protocol. Briefly, both lenses of each animal, each weighing about 8 mg individually, were homogenized in the lysis buffer containing phenol and guanidine thiocyanate. The lysate was then mixed with chloroform and centrifuged. The upper aqueous layer containing the RNAs was then aspirated, thoroughly mixed with 1.5 volumes of ethanol, and transferred quantitatively to an RNeasy mini-column attached to a 2 ml collection tube. After allowing a few minutes for equilibration and RNA binding, the column was spun, filtering out the reagents but retaining the RNA bound on the column. Extraneous material from the column was further removed by washing buffer. The column was then attached to a new collection tube, and RNA was finally eluted by adding RNase-free (50 µl) water to the column and centrifugation. Quantification of the RNA in the elute was then accomplished by measuring absorption at 260/280 nm. The ratio was always more than 1.8. The quality of the RNA was further ascertained electrophoretically. The concentration of RNA in the elute was 350 ng/µl. First strand cDNA synthesis was performed by mixing 1.5 µg (4.3 µl) of the RNA prep with an RT^2^ First Strand cDNA Synthesis kit (SAbioscences, Frederick, MD; MA-03/3311401) reagent containing the primer and the reverse transcriptase in a total volume of 10 µl and incubating for 2 h at 37 °C. The tube was then heated for 5 min at 90 °C to inactivate the reverse transcriptase and chilled on ice; then the volume was increased to 100 µl. This was further diluted to 2,550 µl with 1,275 µl of 2X RT2 SYBR Green qPCR Master Mix (SAbioscences) and 1,175 µl of water. Twenty-five µl of this mixture was then added to each well in the 96-well mi-Finder cassette (MAM 001), containing one universal primer and one gene-specific primer. PCR amplification was performed in an ABI 7900HT Real-Time PCR (Applied Biosystems, Foster City, CA) machine using the three-step cycling program. The resulting threshold cycle (Ct) data for all wells was then transferred to an Excel spreadsheet and exported to Support@SABosciences.com for further analysis.

## Results

As reported previously, using an RT^2^-PCR protocol based on first strand cDNA synthesis by reverse transcriptase reaction followed by quantification of the cDNA by PCR, using the microRNA finder cassette containing the primers corresponding to cDNAs, the presence of all 84 miR species reported to be present in most samples analyzed can be detected (cat no. MAM-001; SA Bioscience). The objective of the present study was to ascertain if incorporating caffeine in a galactose diet has an effect on the miR transcription level. Table 1 summarizes the miR expression levels obtained in the lenses of mice fed a normal diet (Group A), a galactose diet (Group B), and a galactose+caffeine diet C, the expression levels expressed as (2^ (-∆Ct)), and the relative fold (regulation) values. As is apparent by the fold values labeled B/A (the ratio of expression between galactosemic and normal lenses), galactose feeding leads to a substantial elevation in the transcription of at least 19 miRs, the fold value at least more than 2. A similar elevation in most of these miRs has been previously reported [39]. These miRs have been widely reported to be largely proapoptotic. However, the transcription of at least three miRs was intriguingly repressed, suggesting the possibility of a protective tissue response, moderating the extent of tissue damage.

**Table 1:** Both the lenses of the animals were pooled for RNA isolation and subsequent processing for detection of miRs by RT2-PCR.

Name	Group A Normal	Group B Galactose	Group CGal+Caffeine	Fold values (C/A)	Fold values (C/B)	Fold values (B/A)
mmu-miR-32	0.206379	0.028751	0.048312	−4.27	1.68	−7.18
mmu-miR-503	0.215076	0.501	0.932804	4.34	1.86	2.33
mmu-miR-199a-5p	0.005404	0.001846	0.003261	−1.66	1.77	−2.93
mmu-miR-138		0.021333	0.036355		1.70	
mmu-miR-142–5p	0.0012	0.000807	0.000469	−2.56	−1.72	−1.49
mmu-miR-16	4.494815	35.633964	18.978262	4.22	−1.88	7.93
mmu-miR-124		2.653982	1.346003		−1.97	1.83
mmu-miR-126–3p	0.337068	2.86875	1.129979	3.35	−2.54	8.51
mmu-miR-9	0.132899	1.892583	0.666452	5.01	−2.84	14.24
mmu-miR-27a		8.5713	5.320316		−1.61	2.14
mmu-miR-155		0.002994	0.001531		−1.96	2.58
mmu-miR-872	0.51421	3.31476	2.061161	4.01	−1.61	6.45
mmu-miR-126–5p	0.155496	2.671638	1.323574	8.51	−2.02	17.18
mmu-miR-196b	0.00005	0.000807	0.000418	8.36	−1.93	16.14
mmu-miR-196a	0.0003	0.002178	0.001035	3.45	−2.10	7.26
mmu-miR-30c	7.572804	34.424944	22.789992	3.01	−1.51	4.55
mmu-miR-880	0.000425	0.001157	0.000675	1.59	−1.71	2.72
mmu-miR-182	0.046855	0.143981	0.074427	1.59	−1.93	3.07
mmu-miR-411	0.013525	0.074768	0.04618	3.41	−1.62	5.53
mmu-miR-125b-3p		0.002612	0.001555		−1.68	2.08
mmu-miR-295	0.000097	0.000807	0.000239	2.46	−3.38	8.32
mmu-miR-218	4.638208	33.580832	23.433166	5.05	−1.43	7.24
mmu-miR-335–5p	0.032472	1.255721	0.240619	7.41	−5.22	38.67
mmu-miR-101a	0.549524	0.444408	0.215054	−2.56	−2.07	
mmu-miR-141	0.003004	0.001621	0.000889	−3.38	−1.82	−1.85
mmu-miR-374	0.845519	8.645346	5.530468	6.54	−1.56	10.22

The values in the table represent means of 4 lenses in groups A, 6 lenses in group B and 4 lenses in group C. The numbers represent the gene expression levels as (2^(-ΔCt)), normalized with respect to the reagent controls as well as the house keeping gene. The fold changes are ratios of the expression levels.

The fold values summarized under the column C/A represent the effect of incorporating caffeine in the galactose diet. Adding caffeine to the galactose diet significantly annulled the miR elevation caused by galactose. The galactose+caffeine/normal ratios were significantly lower than the fold values for the galactose without caffeine/normal. This conforms to our earlier observation showing that caffeine inhibits galactose-induced apoptosis, while inhibiting the formation of cataracts [46].

MiR expression in the galactose plus caffeine group was compared with that in the galactose alone group under C/B. The relative expression levels of the miRs in the galactose+caffeine group were strikingly lower compared to those in the galactose alone group; the ratios were negative. The results therefore strongly suggest that caffeine inhibits the transcription of toxic miRs, as indicated by the C/B ratios.

## Discussion

The intake of caffeine, derived either through a beverage or as a drug, is followed by several normal physiologic effects, such as stimulating the nervous, cardiovascular, and musculoskeletal systems, relaxing the bronchial and vascular smooth muscles, and promoting diuresis, intestinal motility, etc [50-56]. The stimulation of the nervous system is reflected by enhanced alertness, mental hyperactivity, arousal, wakefulness, enhanced cognitive ability, tolerance of pain, and ergonomic improvements. These neural effects are attributed largely to the competitive binding of caffeine to adenosine receptors on the presynaptic nerve terminals, as the structure of caffeine is similar to adenosine. Since the occupation of these receptors by adenosine limits the amount of calcium translocation to inside the cells through the voltage-gated and voltage-independent calcium channels, their blockade by caffeine minimizes such limitation, and thus increases cytosolic calcium levels [57,58]. The increase in cellular calcium in turn facilitates vesiculation of the neurotransmitters and their release in the synaptic cleft. The release of these neurotransmitters including acetylcholine, norepinephrine, dopamine, and some others [59-61] is thus responsible for enhancing neural transduction and stimulating the cardiac pacemaker cells [62,63]. The cardiovascular stimulation reflected by enhanced contractibility of the cardiac muscle has also been shown to the effect of opening the calcium channels in the sarcoplasmic reticulum of the cardiac myocytes, releasing the sequestered Ca^2+^ into the cytosol. The increased cytosolic Ca^2+^ in turn stimulates muscle contraction via interaction with the actomyosin complex. The vasodilatory effect of Ca^2+^ is exerted through activation of nitric oxide synthase, resulting in increased nitric oxide and cyclic guanosine monophosphate levels. Therefore, most of the acute effects of caffeine are exerted through its action of increasing the intracellular levels of free calcium. In addition, caffeine inhibits cyclic adenosine monophosphate phosphodiesterase, increasing the adenosine triphosphate levels [64]. Although this phenomenon is used by athletes to improve their physical performance, such elevation requires much higher amounts of caffeine than ordinarily possible. Hence, the action of caffeine through the latter effect is normally less significant.

However, in addition to these transient effects, which are largely obliterated in regular coffee drinkers, several recent reports have emphasized many long-term beneficial effects of caffeine consumption, such as caffeine’s effect against the development of age-related dementia and cognitive loss [65-67], as well as Alzheimer disease [68,69]. The latter effect is ascribed to inhibition of the formation and accumulation of beta-amyloid plaques and fibrils in the brain due to enhanced cleavage of the parent transmembrane amyloid precursor protein [70-73]. Caffeine has also been found to be beneficial against Parkinson disease [74,75]. This is linked to caffeine’s preventive effect against oxidative damage to the cells in the pars compacta of the substantia nigra [76-78] and consequent imbalance in the production and release of dopamine and acetylcholine that causes dyskinesia. Caffeine consumption has also been shown to be related to attenuation of liver cirrhosis [79,80] and the development of certain cancers [81,82].

Interestingly, caffeine has a highly significant effect of decreasing the risk of the development of type 2 diabetes [83-85], a highly prevalent aging disease whose incidence is projected to undergo a substantial increase in the coming years [86]. Additionally, age- and diabetes-associated mortality has also been found to be inversely correlated with coffee consumption; mortality is significantly lower in groups consuming higher amounts of coffee per day [87-89]. The mechanism of such protective effects of caffeine could be diversified. The possibility that caffeine could also be related to its continued diuretic effect and consequent excretion of the toxicants produced by oxidative stress, including those produced by oxidation of sugars, and of the sugars themselves, remains to be elucidated. Since caffeine is a potent antioxidant, we propose that in addition to acting as general antioxidant, caffeine also inhibits oxidative stress–induced aberrations in microRNA transcription. Therefore, caffeine can potentially protect tissue against miRNA-induced silencing of important antioxidant genes. This effect of inhibiting oxidation-induced gene silencing likely provides additional antioxidant advantage compared to most other antioxidants such as superoxide dismutase, catalase, and other peroxide decomposing enzymes that remain essentially restricted in the extranuclear compartment of the cell. Most redox active soluble compounds present in cells are also unstable and rapidly catabolized [90]. Caffeine, however, is much more stable and well distributed following its intake. The present findings showing caffeine’s inhibitory effect against oxidative stress–induced elevation of toxic miRs is hence considered pathophysiologically useful. The findings also suggest that the inhibitory effect of caffeine against apoptosis that precedes sugar cataract formation as reported earlier [46] is due to inhibition of the biogenesis of proapoptotic miRs. Although caffeine’s effect of preventing oxidative stress and biogenesis of toxic miRs has so far been shown in hyper-galactosemic conditions, we speculate caffeine has similar effects against the effects of high glucose that persist in diabetic conditions. The elevation of microRNAs, in addition to causing gene silencing at the level of mRNAs involved in the biogenesis of antioxidant enzymes, could also exert a detrimental effect by inhibiting certain upstream pathways such as the nuclear factor (erythroid-derived 2)-like 2/Kelch-like ECH-associated protein 1 pathway [91,92] involved in the transcription of antioxidant genes. Further studies on the mode of caffeine effects are thus in progress.

According the U.S. Food and Drug Administration, caffeine, one of the most common nutraceuticals, is highly safe for human use. Caffeine’s median lethal dose (LD50) is estimated to be at least greater than 10 g, with lethality unknown. In experimental animals, the LD50 is high: 355 mg/kg (Merck Index) in rats, equivalent to about 25 g/70 kg in humans. The effective anticataract dose of caffeine in galactosemic rats is 15 mg/kg/day [46], which is only about 4% of the LD50. No toxicity, systemic or ocular, was observed. Additionally, several multivariate analyses identifying the nutritional risk factors for cataracts have also found caffeine to be safe. Indeed, as summarized before, the global incidence of cataracts [48] also appears to be negatively correlated with caffeine intake, being markedly lower in countries with higher intake (≥200 mg per day) than in countries with lower intake such as China and India and in Central and South America [49]. Contrary to some early suggestions [93] that caffeine could have an adverse effect of increasing intraocular pressure, substantially large epidemiological and clinical studies [94-97] have disproved this. A transient rise of 1–2 mmHg sometimes noted following a coffee drink is in fact attributable to the hemodynamic effect due to water content associated with the drink [98-101], water intake being a standard test for glaucoma. Therefore, the possibility of any significant toxic effect of caffeine to eye is highly remote. The results showing its effectiveness against the transcription of several pro apoptotic microRNAs, coupled with its inhibitory effect against actual cataract formation in vivo strongly suggests its possible pharmacological use in cataract prevention. The inhibitory effect of this compound against toxic miRNA transcription has been shown for the first time.
